# Analysis of abnormal muscle activities in patients with loss of cervical lordosis: a cross-sectional study

**DOI:** 10.1186/s12891-023-06782-3

**Published:** 2023-08-22

**Authors:** Jiwoon Lim, Dajeong Lee, Sangyoung Kim, Seungeun Lee, Ju Seok Ryu

**Affiliations:** 1 Department of Rehabilitation Medicine, Korea University Ansan Hospital, Ansan-si, South Korea; 2 Department of Medicine, Seoul National University College of Medicine, Seoul, South Korea; 3 Department of Rehabilitation Medicine, Seoul National University Hospital, Seoul, South Korea; 4 Department of Rehabilitation Medicine, Seoul National University Bundang Hospital, Seoul National University College of Medicine, 82 Gumi-Ro 173 Beon-Gil, Bundang-Gu, Seongnam-Si, Gyeonggi-Do 13620 South Korea; 5 Department of Rehabilitation Medicine, Seoul National University College of Medicine, Seoul, South Korea

**Keywords:** Lordosis, Kyphosis, Trapezius, Muscle activity

## Abstract

**Background:**

This study aimed to detect the differences in cervical muscle activation patterns in people with versus without cervical lordosis and explore the possible mechanism of cervical pain originating therein.

**Methods:**

This cross-sectional design included 39 participants without and 18 with normal cervical lordosis. Muscular activation was measured for 5 s in both groups using surface electromyography. Subsequently, the root mean square (RMS) of muscle amplitude was obtained at the bilateral splenius capitis, upper and lower parts of the splenius cervicis, upper and lower parts of the semispinalis cervicis, sternocleidomastoid, upper trapezius, and rhomboid muscles in five cervical positions: 0° (resting), 30° of flexion, 30° of extension, 60° of extension, and upon a 1-kg load on the head in a resting posture.

**Results:**

The RMS values of the upper trapezius muscle at all postures and the rhomboid muscles at 60° of extension were significantly lower in the loss of lordosis than control group. Comparing the RMS ratio of each posture to the resting position, the ratio of the upper trapezius at flexion was significantly higher and that of the rhomboids at 60° of extension and upon loading was significantly lower in the loss of lordosis than control group. Moreover, the pattern changes in the RMS values according to posture showed a similar shape in these two muscles, and lower in the loss of lordosis than the normal group.

**Conclusions:**

The loss of normal cervical alignment may correlate with predisposed conditions such as reduced muscle activation of the trapezius and rhomboid muscle, and may also provoke over-firing of the upper trapezius muscle, possibly increasing neck musculoskeletal pain.

Trial registration.

Clinicaltrials.gov, registration number: NCT03710785.

**Supplementary Information:**

The online version contains supplementary material available at 10.1186/s12891-023-06782-3.

## Background

Cervical lordosis is defined as the normal inward curvature of the cervical spine. Loss of cervical lordosis is associated with neck pain, headache, and reduced quality of life [1, 2]. This altered curvature of the cervical spine induces further axial load and results in the progression of kyphotic posture [3]. Cervical kyphosis, the most common deformity affecting the normal functioning of cervical spine, causes significant disability in affected patients. Cervical kyphosis can be regional or global and is associated with reduced quality of life [4].

However, due to a sedentary lifestyle, an increasing number of people are losing this normal curvature and suffering from cervical straightening and kyphosis [5]. The slouched sitting posture, which is often reported in people who sit for a long time, causes thoracic kyphosis and more muscle activation in the neck and shoulder regions [6, 7]. A slouched posture is often characterized by a combination of forward head posture. In particular, prolonged use of computers and smartphones may require maintaining a continuous forward head posture in users [8]. In a previous study, it was reported that screen view time is associated with increased neck and head flexion posture in children, especially in a sitting position [9]. Use of a smartphone in a greater flexed neck posture resulted in a larger biomechanical burden on kinematics and neck muscle loading, which may increase the risk of cervical and shoulder musculoskeletal injuries [10].

Previous studies have been documented that forward had postures can be associated with abnormalities in neuromuscular function, such as reduced activation of the deep muscles of the cervical spine, as well as over-activity of the superficial neck muscles in people with chronic neck pain [11–13]. One electromyographic study has identified the correlation between neck acceleration and muscle activation in adults with neck pain [14]. However, several previous studies that examine head posture using electromyography (EMG) have focused on specific one neck muscle. Moreover, there has few reports regarding a relationship between muscle contraction, head posture, and cervical curvature using EMG. No study has revealed whether the cervical muscles affect the maintenance of cervical lordosis and confirmed the relation of pain in these patients.

In this study, we hypothesized that the loss of physiological lordosis of the cervical spine alters the direction of cervical muscular contractions, resulting in an abnormal contractile pattern such as over-firing. Moreover, excessive abnormal contractions of these muscles can also cause cervical pain. Therefore, we investigated the differences in the activation patterns of the cervical muscles between people with and without cervical lordosis.

## Methods

### Ethical approval

The study protocol was approved by Seoul National University Bundang Hospital’s institutional review board (B-1709/421–005) and was registered on clinicaltrials.gov (registration number: NCT03710785, registration date: 18/10/2018). All participants provided written informed consent prior to participation. Their data and safety were monitored every 6 months.

### Study design and participants

This cross-sectional study was conducted from October 23, 2017, to June 30, 2020. During the screening process, we recorded the demographic data and medical history of each participant, and performed a thorough physical examination. All included participants were over 19 years old and agreed to provide informed consent. The study population was further divided into two groups to investigate the cervical curvature on lateral radiographs: participants with loss of lordosis (LL group) and participants with maintained lordosis (normal group). The exclusion criteria were as follows: (1) history of central nervous system diseases, (2) neuromuscular diseases, (3) spinal abnormalities, (4) structural disorders of the cervical spine, (5) history of cervical trauma or inflammatory rheumatic diseases, and (6) cervical surgery or exercise rehabilitation programs.

### Measurements

#### Clinical evaluation [15]

Clinical evaluations included a test for the presence of pain, pain intensity at rest (numeric rating scale [NRS], score 0–10), weekly exercise frequency and duration (hours per week), daily usage time of computer and smartphone (hour), sleep time (hour), sleep quality (0–100), and quality of life questionnaire using the Korean version of the European Quality of Life Five-Dimension-Five-Level (EQ-5D-5L). The EQ-5D-5L is the most widely used indirect evaluation tool for health state, including five dimensions (mobility, self-care, usual activities, pain/discomfort, and anxiety/depression). Each domain is scored on a scale of 1–5 [16]. We used the questionnaire of the Korean-adapted version, which has been shown to be reliable and valid [17].

#### Radiographic evaluation

Lateral radiographs were obtained with each subject standing in an erect position with one shoulder contacting the detector holder, maintaining horizontal gaze (patients looked at the point on the wall at the eye level in front of them), slightly flexing the elbows, and relaxing the shoulder and arms at the side of their body. The subject slightly extends the chin to prevent overlap between the jaw and cervical spine. Hips and knees were in full extension.

**Fig. 1:**
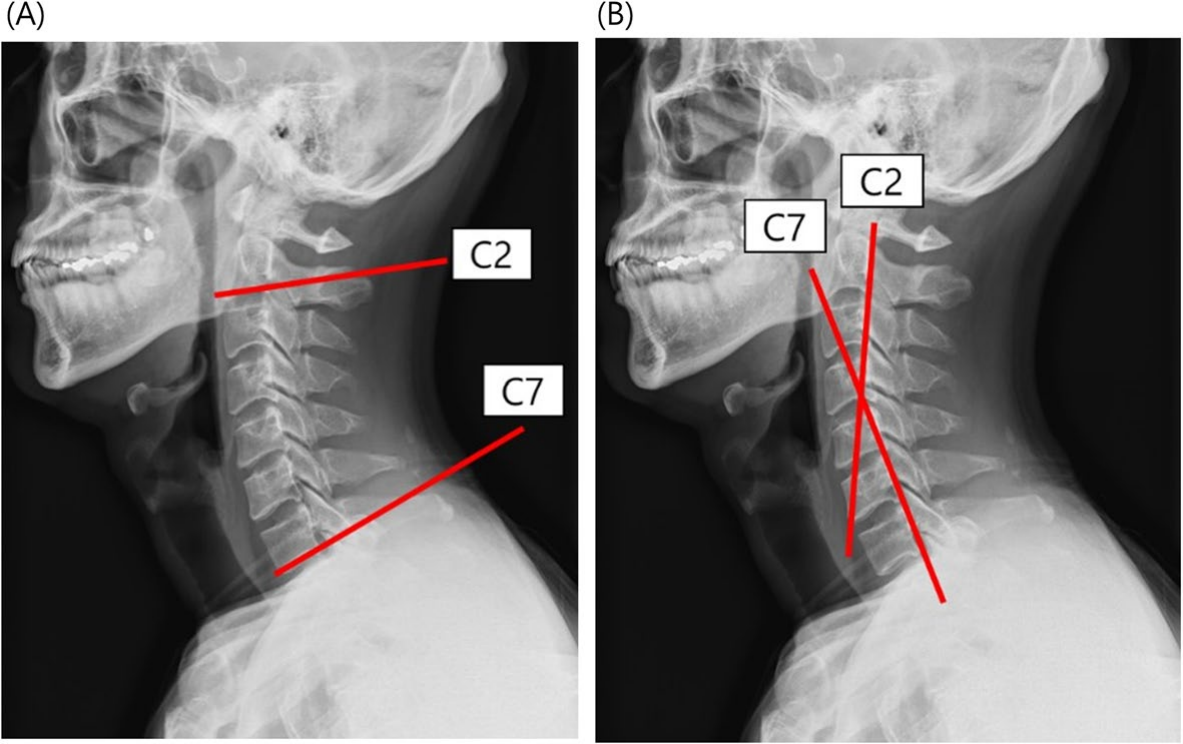
Sagittal alignment of the cervical spine was measured by modified Cobb’s method (mCM) and posterior tangent method (PTM). The mCM measures the angle between the lines parallel to the C2 inferior endplate and C7 inferior endplate (Fig. 1 (**A**)), and the PTM measures the angle between the lines parallel to the C2 posterior body and C7 posterior body (Fig. 1 (**B**))

Sagittal alignment of the cervical spine was measured using the modified Cobb’s method (mCM) and the posterior tangent method (PTM) [18, 19]. The mCM measures the angle between the lines parallel to the C2 inferior endplate and C7 inferior endplate (Fig. 1(A)), and the PTM measures the angle between the lines parallel to the C2 posterior body and C7 posterior body (Fig. 1(B)). In previous studies, mCM and PTM have shown excellent intra- and inter-observer reliabilities for measuring cervical sagittal alignment [20, 21]. Using these methods, we defined the normal range of cervical lordosis as -34.6° to -6.0° measured by the mCM and -25.8° to -1.4° measured by the PTM using average ± 1 standard deviation. [22, 23] Then, we allocated the participants to the normal group when the mCM and PTM measurements were within the normal range and to the loss of lordosis (LL) group when at least one of the measurements by the mCM or PTM were out of the normal range.

Radiographic evaluations were performed manually by two blinded examiners who had independently reviewed and measured, and the average values were used in our study.

### Surface EMG signal acquisition

The participants positioned their hips and knees at a 90 degree, feet positioned shoulder width apart with relaxed at the side of body. They were asked to look straight ahead at eye level, keeping natural head position and instructed to maintain an upright sitting posture to exclude the effect of thoracic kyphosis. [24] This was the initial resting posture. Then, maintaining the reference line always parallel to the imaginary horizontal line between both tragus, measurement postures were determined using a goniometer based on the line connecting the tragus and nose tip. The measurements were performed using a goniometer at five postures: 0° (resting), 30° of flexion, 30° of extension, 60° of extension, and with a 1-kg load on the head in a resting posture.

To evaluate the degree of muscular activation during diverse postures, a wireless eight-channel surface electromyography (S-EMG) analysis system (BTS FREEEMG 1000 with EMG-BTS EMG Analyzer; BTS Bioengineering Co., Milan, Italy) was used for electrophysiological quantitative analysis. The data analysis was performed using a laptop equipped with an EMG analyzer program. To reduce the skin resistance, we wiped the skin with alcohol on the electrode attaching area. The acquisition frequency of the EMG signal was 1024 Hz, and a bandpass filter between 20 and 500 Hz was used.

**Fig. 2:**
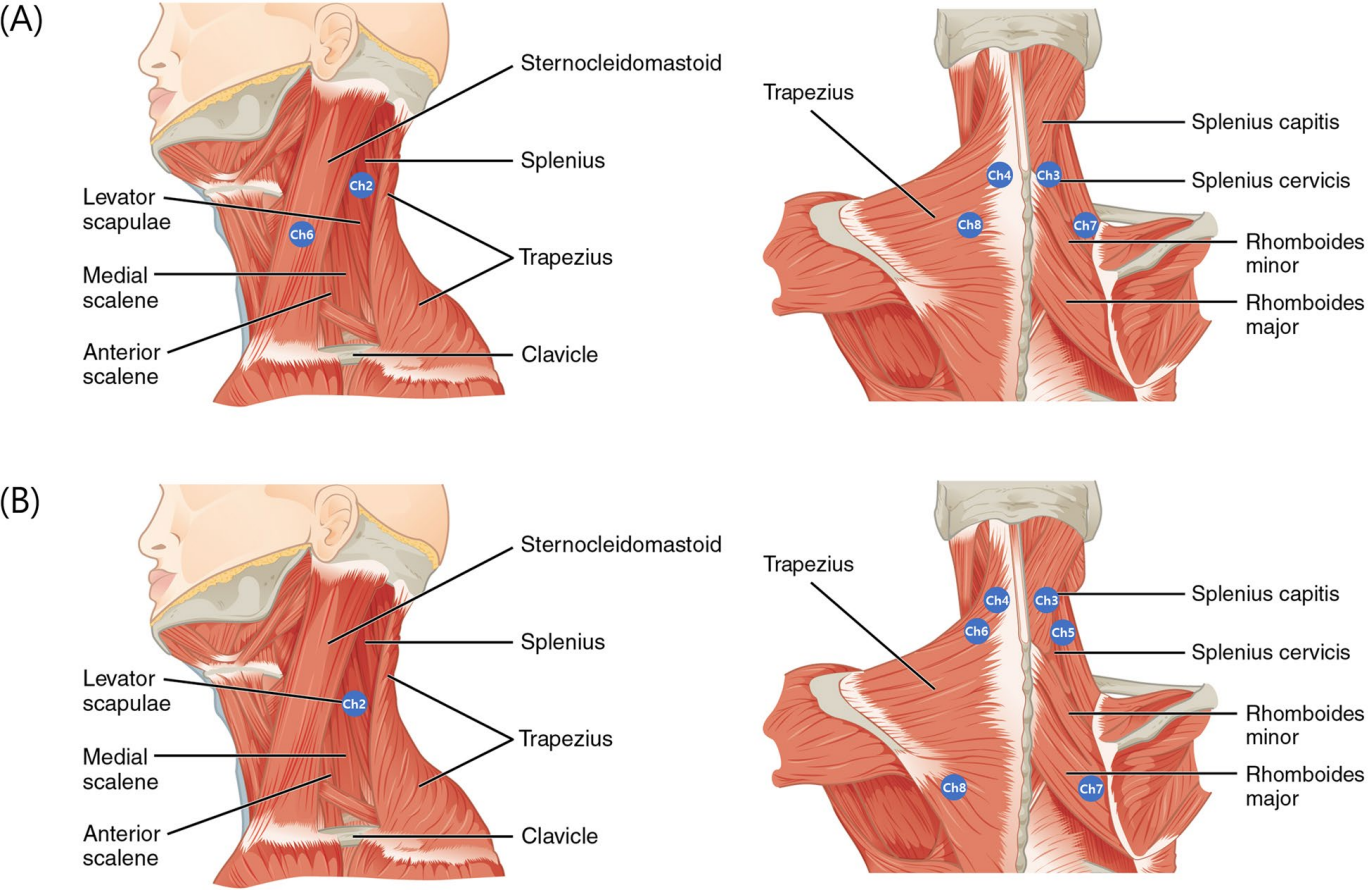
Location of S-EMG electrodes for both trials (**A**) First trial (Ch 1, 2: bilateral splenius capitis, Cha 3, 4: bilateral lower semispinalis cervicis, Ch 5, 6: bilateral sternocleidomastoid, Ch 7, 8: Bilateral upper trapezius/ levator scapulae muscles) (**B**) Second trial (Ch 1, 2: bilateral upper splenius cervicis, Cha 3, 4: bilateral upper semispinalis cervicis, Ch 5, 6: bilateral lower splenius cervicis, Ch 7, 8: Bilateral middle trapezius/ rhomboid muscles). The odd/even number of channels means the right/left side. The copyright owner is OpenStax College (Source: https://cnx.org/contents/FPtK1zmh@8.108:y9_gDy74@5). JL recreated the drawing by adding electrodes to the original drawing. Ch: Channel

Since S-EMG electrodes consisted of eight channels, we evaluated eight sites of the following bilateral muscles in two consecutive trials: the splenius capitis, upper and lower parts of the splenius cervicis, and upper and lower parts of the semispinalis cervicis, sternocleidomastoid, upper trapezius, and rhomboids. As there were few references for the attachment of S-EMG electrodes at the cervical muscles, we evaluated the muscles with ultrasound to determine the most appropriate locations in five adults. The electrode attachment sites were determined to be above the muscle belly, and the crosstalk effect was minimized. The muscles in each trial were allocated to provide the best convenience and accuracy, as the close proximity of some muscles made concurrent measurements difficult. The channels for both trials and the anatomical criteria for the attachment site of the S-EMG electrodes are shown in Fig. 2 and Supplementary Table 1. S-EMG measurements were performed for 5 s at plateau, and then the root mean square (RMS; expressed in microvolts) of the muscle amplitude was obtained. Each module was measured twice at 20-s intervals [25]. Because the S-EMG measurements were performed twice on both sides in each trial, the mean values were used.

### Statistical analysis

The calculation of the sample size was based on a preliminary data in this study, the surface electromyography data of upper trapezius muscle in two groups, prior to main data analysis. Our preliminary S-EMG data of upper trapezius muscle in two groups showed 5.8 ± 3.62 in the loss of lordosis group and 15.9 ± 13.86 in the normal group. With an α of less than 0.05 in the two-tailed tests and a power of 80%, the target sample size of each group was 34 patients (17 in each group). Considering a dropout rate of 5%, we targeted an enrollment of 36 patients.

All statistical analyses were performed using STATA 15.0 (Stata Corporation, College Station, TX, USA) and SPSS version 25d (SPSS Inc., Chicago, IL, USA). The normality of the data was checked with the Kolmogorov–Smirnov test. All demographic data and initial measurements between the two groups were compared using the Mann–Whitney U test for continuous variables and the chi-square test for categorical variables. A linear mixed model was used to compare the RMS values between the two groups according to the eight sites. An adjusted model for age and gender was also assessed. For comparisons of RMS values between the two groups according to the five different postures, two-way repeated measures analysis of variance was used. When the data did not follow the normal distribution, the Mann–Whitney U test was used for comparisons at five diverse postures between the two groups, and the Friedman test was used between the postures in each group. For post-hoc analysis, the Wilcoxon signed-rank test with Bonferroni’s correction was used for multiple comparisons. Additionally, the RMS ratios of the evoked postures compared to the resting posture were calculated and compared between the two groups. Statistical significance was set at *P* < 0.05.

## Results

Fifty-seven participants (39 in the LL group, 18 in the normal group) were enrolled in this study. None of the patients were lost during the experiment. Their demographic data are presented in Table 1. There were no significant differences in age, height, weight, sex, or prevalence of pain between the two groups; however, the intensity of pain was significantly greater in the LL group (*P* = 0.041). The mCM and PTM measurements were 2.1 ± 6.3° and 1.1 ± 9.1° in the LL group and -14.2 ± 6.3° and -17.4 ± 7.3° in the normal group (*P* < 0.05), respectively.

**Table 1:** Demographic data of the normal and loss of lordosis (LL) groups

	LL Group (*n* = 39)	Normal Group (*n* = 18)	*p*-value
Age (years)	33.5 (12.7)	38.7 (14.6)	0.177
Height (cm)	167.1 (7.9)	166.0 (8.4)	0.700
Weight (kg)	59.4 (15.0)	61.5 (8.8)	0.667
Gender (Men/Women)	16/23	10/8	0.306
Modified Cobbs method (°)	2.1 (6.3)	-14.2 (6.3)	0.000*
Posterior tangent method (°)	1.1 (9.1)	-17.4 (7.3)	0.000*
Pain (Y/N)	31/8	12/6	0.296
Pain intensity (0 ~ 10)	6.1 (2.0)	4.5 (1.9)	0.041*
Exercise time, weekly (hr)	0.7 (0.8)	1.0 (0.8)	0.347
Computer use, daily (hr)	3.9 (3.5)	5.2 (3.7)	0.460
Smartphone use, daily (hr)	3.3 (3.7)	4.2 (3.2)	0.311
Sleep time (hr)	6.5 (1.0)	5.9 (1.2)	0.144
Sleep quality (0 ~ 100)	62.8 (15.3)	61.4 (23.6)	0.935
EQ-5D-5L (0 ~ 5)			
Mobility	1.6 (1.0)	1.3 (0.8)	0.527
Pain/discomfort	3.0 (1.0)	2.7 (0.8)	0.631
Self-care	1.3 (0.7)	1.2 (0.4)	0.980
Anxiety/depression	2.2 (1.2)	2.0 (0.9)	0.980

Values are expressed as mean (SD)

^*^p value < 0.05

**Fig. 3:**
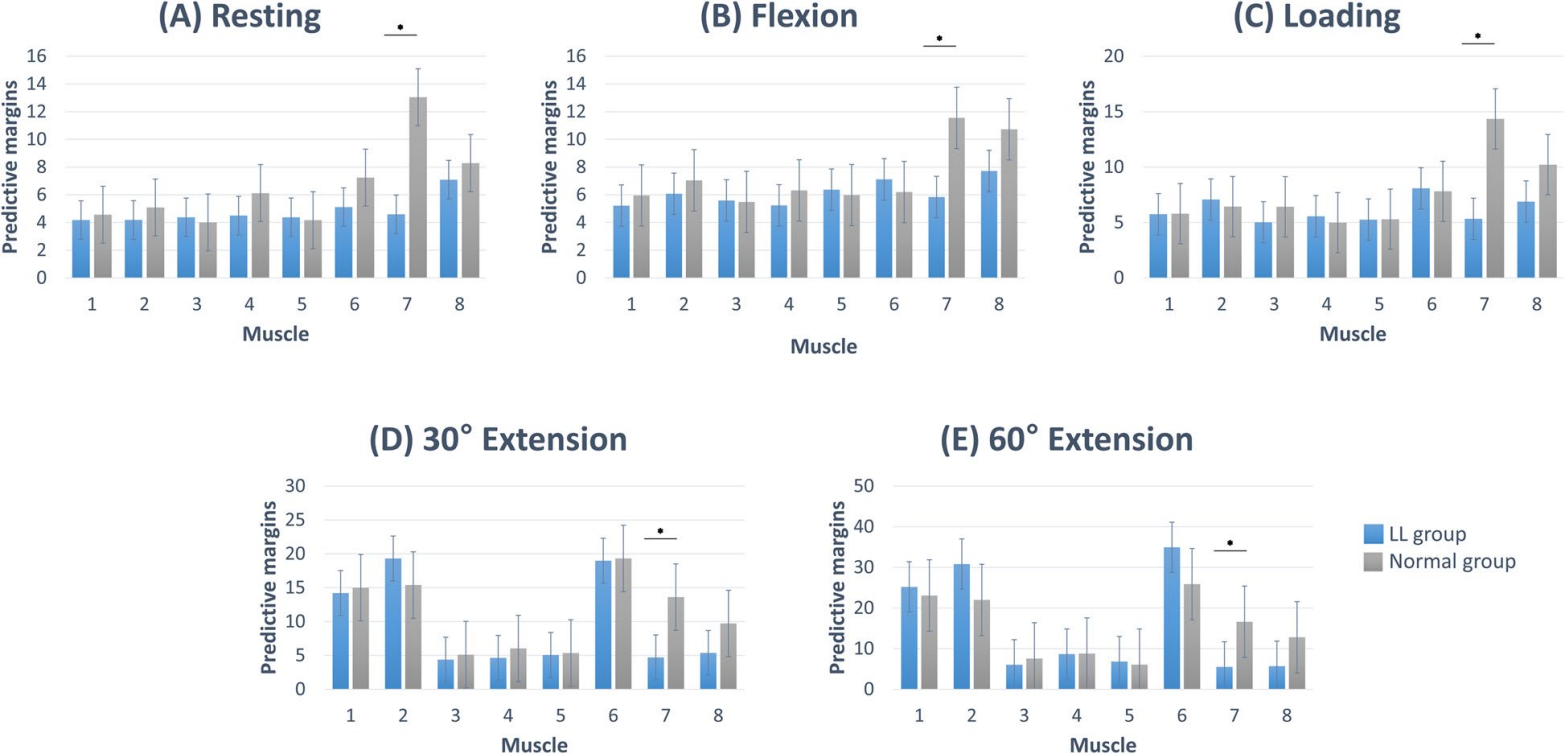
Comparison of eight muscles [1. Splenius capitis muscle, 2. Upper splenius cervicis muscle, 3. Upper semispinalis cervicis muscle, 4. Lower splenius cervicis muscle, 5. Lower semispinalis cervicis muscle, 6. Sternocleidomastoid muscle, 7. Upper trapezius muscle, 8. Rhomboid muscles] between the two groups at specific postures [(**A**) Resting (0°), (**B**) 30° of flexion, (**C**) 1-kg load, (**D**) 30° of extension (**E**) 60° of extension]. The bar graph indicates the predicted margins from adjusted linear mixed models, and error bars indicate 95% CIs. At all postures, the RMS values of the upper trapezius muscle were significantly different (*P* < 0.05). LL: Loss of lordosis

Comparison of the muscles between the two groups revealed that the upper trapezius was significantly different from the other muscles (*P* < 0.05, Fig. 3). In the adjusted model for age and sex, age had a significant influence on the results at 30° flexion (*P* < 0.05), while sex significantly influenced the results at 30° extension (*P* < 0.001) and 60° extension (*P* < 0.01). In the other postures, age and sex did not significantly influence the results.

Comparison of the RMS values of the muscles at five different postures between the two groups, only the upper semispinalis cervicis followed the normal distribution. The RMS values of most of the tested muscles differed according to different postures compared to the resting posture in each group.

**Fig. 4:**
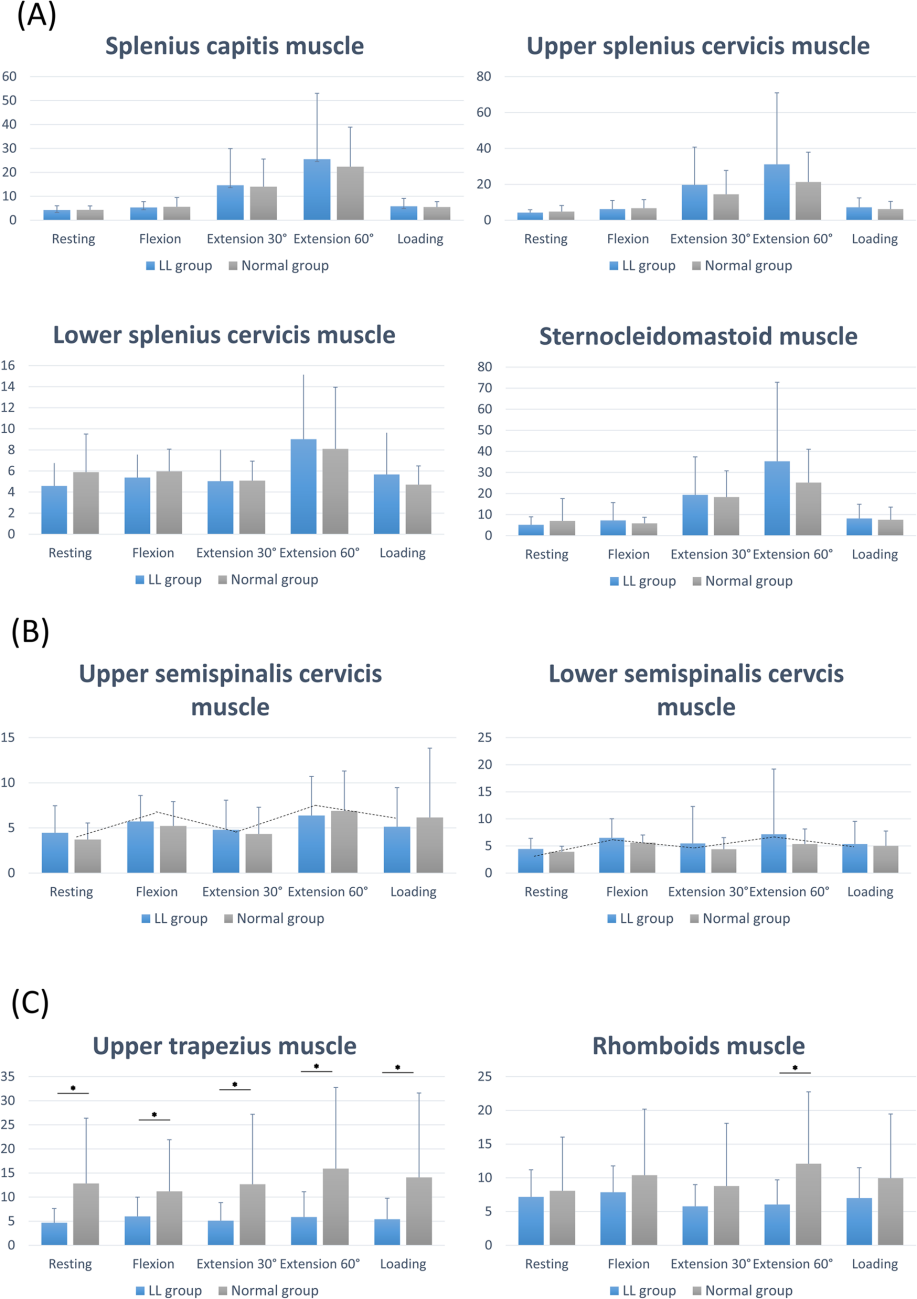
Comparisons of five postures between the two groups at specific tested muscles. The RMS values of the splenius capitis, the upper and lower part of the splenius cervicis, and sternocleidomastoid muscles showed similar shape which had a peak at 60° extension posture (4th) and a relatively higher RMS value in the loss of lordosis group (**A**), the upper and lower part of the semispinalis cervicis muscles showed similar shape (M shape) and a relatively higher RMS value in the loss of lordosis group (**B**), and the upper and rhomboid muscles showed similar shape and significantly lower RMS values at all postures in the loss of lordosis group than the normal group (**C**) (*P* < 0.05)

The pattern of changes in RMS values according to posture in the splenius capitis, upper and lower parts of the splenius cervicis, and sternocleidomastoid muscles showed similar shapes, with a peak at 60° extension and a relatively higher RMS value in the LL group than in the normal group; however, the value was not statistically significant (Fig. 4(A)). The upper and lower parts of the semispinalis cervicis muscles showed a similar shape (M shape) and a relatively higher RMS value in the LL group than in the normal group; however, the difference was not statistically significant (Fig. 4(B)). The upper trapezius muscle and rhomboid muscle exhibit similar shapes. Unlike the other muscles, the RMS values of the upper trapezius muscle at all postures and the rhomboid muscles at 60° extension were significantly lower in the LL group than in the normal group (*P* < 0.05, Table 2, Fig. 4(C)).

**Table 2:** The surface electromyography raw data of cervical muscles at different postures in the loss of lordosis (LL) and normal groups

	Resting			Flexion			Extension 30°			Extension 60°			Loading		
	LL group	Normal group	*P* value	LL group	Normal group	*P* value	LL group	Normal group	*P* value	LL group	Normal group	*P* value	LL group	Normal group	*P* value
Splenius capitis muscle	4.26 (1.82)	4.34 (1.63)	0.67	5.36 (2.44)	5.61 (3.87)	0.84	14.60 (15.33)	14.05 (11.50)	0.60	25.52 (27.51)	22.36 (16.53)	0.70	5.86 (3.28)	5.54 (2.25)	0.93
Upper splenius cervicis muscle	4.27 (1.56)	4.86 (3.32)	0.80	6.22 (4.78)	6.69 (4.75)	0.43	19.71 (21.00)	14.45 (13.31)	0.64	31.16 (39.84)	21.29 (16.64)	0.93	7.18 (5.28)	6.16 (4.33)	0.45
Upper semispinalis cervicis muscle	4.46 (3.00)	3.72 (1.83)	0.36	5.72 (2.86)	5.22 (2.69)	0.40	4.79 (3.29)	4.33 (2.96)	0.60	6.37 (4.33)	6.88 (4.43)	0.60	5.13 (4.33)	6.15 (7.68)	0.91
Lower splenius cervicis muscle	4.58 (2.17)	5.90 (3.61)	0.15	5.38 (2.18)	5.97 (2.10)	0.24	5.04 (2.98)	5.08 (1.85)	0.28	9.01 (6.13)	8.10 (5.85)	0.81	5.67 (3.84)	4.71 (1.78)	0.79
Lower semispinalis cervcis muscle	4.46 (1.96)	3.94 (1.02)	0.63	6.51 (3.52)	5.63 (1.41)	0.78	5.48 (6.81)	4.41 (2.13)	0.95	7.16 (12.05)	5.36 (2.79)	0.81	5.37 (4.18)	5.02 (2.75)	0.63
Sternocleidomastoid muscle	5.18 (3.79)	7.02 (10.56)	0.37	7.25 (8.44)	5.85 (2.85)	0.62	19.39 (18.02)	18.36 (12.42)	0.68	35.28 (37.50)	25.18 (15.83)	0.67	8.19 (6.74)	7.55 (6.03)	0.98
Upper trapezius muscle	4.67 (2.97)*	**12.82 (13.56)**	**0.004**	**5.98 (4.01)**	**11.20 (10.70)**	**0.03**	5.10 (3.75)*	**12.66 (14.52)**	**0.003**	5.86 (5.24)*	**15.91 (16.83)**	**0.003**	5.41 (4.35)*	**14.08 (17.53)**	**0.005**
Rhomboids muscle	7.18 (4.01)	8.06 (7.97)	0.67	7.86 (3.91)	10.38 (9.81)	0.55	5.78 (3.21)	8.77 (9.31)	0.63	6.04 (3.64)*	**12.08 (10.67)**	**0.04**	7.00 (4.49)	9.95 (9.50)	0.08

Values are expressed as mean (SD)

^*^p value < 0.05

When we calculated the ratio of 30° flexion, 30° extension, 60° extension, and loading postures to the resting posture, and compared these values between the two groups, the ratio of the upper trapezius muscle in the flexion posture was significantly higher in the LL group than in the normal group (*P* < 0.05, Table 3). The ratio of rhomboid muscles at 60° extension and loading posture to resting posture was significantly lower in the LL group than in the normal group (*P* < 0.005, Table 3). Although the differences were not statistically significant, a similar pattern was observed in the 30° extension posture (*P* = 0.05, Table 3).

**Table 3:** The surface electromyography ratio values compared to resting position in the loss of lordosis (LL) and normal groups

	Flexion		Extension 30°		Extension 60°		Loading	
**Ratio**	LL G	Normal G	LL G	Normal G	LL G	Normal G	LL G	Normal G
Splenius capitis muscle	1.34 (0.59)	1.52 (1.54)	3.80 (3.97)	3.94 (4.55)	6.33 (8.24)	6.39 (6.34)	1.48 (1.19)	1.42 (0.80)
Upper splenius cervicis muscle	1.43 (0.91)	1.69 (1.45)	5.00 (5.78)	3.26 (2.46)	7.66 (12.38)	5.15 (4.56)	1.77 (1.47)	1.39 (0.59)
Upper semispinalis cervicis muscle	1.41 (0.42)	1.47 (0.50)	1.13 (0.40)	1.19 (0.37)	1.43 (1.01)	1.94 (1.11)	1.13 (0.48)	1.21 (0.58)
Lower splenius cervicis muscle	1.22 (0.24)	1.17 (0.40)	1.11 (0.35)	1.03 (0.44)	2.15 (1.99)	1.72 (1.40)	1.17 (0.35)	0.93 (0.24)
Lower semispinalis cervcis muscle	1.47 (0.38)	1.46 (0.25)	1.28 (1.68)	1.18 (0.68)	1.58 (3.06)	1.41 (0.84)	1.19 (0.73)	1.37 (0.89)
Sternocleidomastoid muscle muscle	1.36 (0.70)	1.22 (0.67)	4.38 (4.73)	3.75 (2.72)	7.80 (11.20)	6.24 (5.74)	1.76 (1.45)	1.63 (1.16)
Upper trapezius muscle	1.32 (0.46)^*^	**1.04 (0.42)**	1.16 (0.79)	1.19 (0.69)	1.23 (0.79)	1.69 (1.97)	1.17 (1.13)	1.37 (1.28)
Rhomboids muscle	1.15 (0.29)	1.35 (0.47)	0.87 (0.27)	1.14 (0.60)	0.88 (0.48)*	**1.64 (1.20)**	**1.09 (0.91)**	**1.30 (0.35)**

*G* Group

^*^p value < 0.05

## Discussion

This study was designed to investigate the association between cervical muscular activation patterns and cervical lordosis in diverse postures. Although not all neck extensor muscles showed a significant difference between the RMS values in the normal and LL groups, the overall tendency of muscle activity showed three distinctive patterns. The first pattern included the upper trapezius and rhomboid muscles, which showed lower RMS values in all postures in the LL group compared to the normal group. Lower RMS values imply less muscle activation in all postures in patients with loss of normal lordosis. In particular, as the resting posture does not require strong muscular contractions, we reasoned that the lower RMS values at the resting posture in the LL group could be correlated muscle weakness, weak movement demand, or small muscle bulk. Although weaker muscles tend to overfire to make a movement in general, they showed significantly lower RMS values during flexion, extension, and loading postures. This could be due to the adaptation (chronic disuse) of loss of cervical lordosis and reduced use of the trapezius during diverse postures. Under such circumstances, it is difficult to determine whether the weak muscle demand of the trapezius led to the development of kyphotic change and secondary disuse atrophy of the muscle, or vice versa.

The second pattern, showing a peak at 60° extension posture, was found in large muscles with a long lever arm, such as the splenius capitis, upper splenius cervicis, lower splenius cervicis, and sternocleidomastoid muscles (Fig. 4(A)). Although the differences were not statistically significant, this pattern showed lower or similar RMS values at rest and increased RMS values in dynamic neck postures in the LL group compared to the normal group. The splenius muscles originate from the spinous process and insert onto the mastoid process, and the sternocleidomastoid muscle also has a long lever arm connecting the sternum and clavicle to the mastoid process. We speculate that the directions in which these muscles act change when cervical lordosis is lost and kyphosis develops, especially in muscles with a relatively long lever arm and in those connecting the skull and cervical spine or sternum. Therefore, compared to the normal group, the long paraspinal muscles in the LL group showed increased RMS values in evoked postures than in the resting posture (overfiring). In accordance with our findings, a previous study confirmed increased activation in the sternocleidomastoid and muscle in the forward flexed posture [12]. However, a prospective follow-up study with a calculated sample size or stricter inclusion criteria is required to verify the statistical significance of our findings. The third pattern was M-shaped and was found in the upper and lower parts of the semispinalis muscles. Anatomically, these muscles originate and insert onto the cervical spine, are relatively small, and act at short segment level. The activation pattern according to the postures was minimal in lordosis or kyphosis.

Moreover, in the 30° flexion posture, the ratio of the upper trapezius muscle to the resting posture in the LL group was significantly higher than that in the normal group (Table 3). Considering the significantly lower RMS value of the upper trapezius in the LL group in the resting posture, the increase implies overfiring in the 30° flexion posture. In this posture, the center of the head moves anteriorly, and the movement arm length increases, thus creating a larger bending moment [19]. The resultant larger bending moment requires greater paraspinal muscle contraction to keep the head erect, which in turn can cause muscle fatigue and pain. It is also known that the fatiguing contractions may lead to muscle pain [26]. Considering that the flexion posture has the same biomechanical aspect as kyphosis, muscular overfiring can be a cause of cervical pain of the upper trapezius muscle in patients with loss of cervical lordosis. In addition, kyphotic cervical deformities shift the axial load anteriorly, potentially accelerating cervical disc degeneration [19]. In the present study, the severity of neck pain was significantly greater in the people without normal lordosis.

In extension and loading postures, the ratio of the rhomboid muscle compared to resting posture in patients with loss of cervical lordosis was lower than that in the normal group (Table 3). The rhomboid muscles act as a scapular retractor that is important for the stability of both the shoulder girdle and scapula. The results obtained in our survey indicate that loss of cervical lordosis is closely related to impaired muscle performance of the scapular retractors, which may lead to slouched forward shoulders [6].

Previous studies using cross-sectional analysis through magnetic resonance imaging examinations have demonstrated that patients with cervical straightening show atrophy of the cervical extensor muscles [27, 28]. Additionally, our study showed that the muscle activity, which directly reveals the quality of the muscle, is decreased in patients with loss of cervical lordosis. Since a recent study has demonstrated that lordosis improves after neck strengthening exercises, the association between cervical muscles and the alignment seems clear [15, 29]. However, a follow-up study to measure the size of upper trapezius muscle in patients with cervical kyphotic change is warranted.

The numerous methods of neck strengthening exercises have been studied to restore physiological cervical alignment. However, it may accelerate disc degeneration, especially in the upper cervical region [29, 30]. Therefore, in accordance with our previous study [15], the modified cervical and scapular retraction exercise, which involves strengthening the shoulder retraction muscles but excludes neck muscle strengthening exercises that could potentially damage the discs, is helpful in patients with loss of cervical lordosis and neck pain. Moreover, the habit of positioning the head forward during daily activities, such as using computers, reading a book, or driving, can stress the cervical spine and lead to the loss of physiological lordosis. It was thought that maintaining proper posture according to normal cervical alignment is considered important.

This study had some limitations. First is the crosstalk phenomenon, which is an intrinsic limitation of S-EMG. As electrodes are attached to the skin, the electric current generated by nearby muscles can interfere with the signals from the targeted muscle. However, to overcome this limitation, electrodes were attached to the same muscle belly, and we focused only on the differences in signals between various postures, offsetting the muscle crosstalk phenomenon. Second, we did not measure maximum voluntary isometric contraction (MVIC) and normalization. Normalization of EMGs is necessary because of the many technical, anatomical, physiological, sex, and investigator factors that can influence the magnitude of EMG [31, 32]. In the present study, we used the ratios of the evoked postures compared to the resting posture instead of using MVIC ratio and compared the changes in each participant. However, to compare the EMG activity between groups accurately, further studies should include the normalization of the EMG signals. Third, the normal range of cervical lordosis is diverse. There is no consensus regarding the normal value of the cervical curvature does not exist. In addition, most studies using the PTM usually apply -4°–4° as cervical straightening; [18, 33] however, this value was decided arbitrarily “based on the typical error of measurement from radiographic segmental angles” [18]. In the present study, we used the average value ± 1 standard deviation as the normal cervical lordosis considering the overlaps [22, 23]. For better evaluation, a guideline on the normal range of cervical lordosis is considered essential for future research. Fourth, the gender ratio was different between the two groups. However, the adjusted linear mixed model for age and sex showed similar results.

## Conclusions

It is suggested that the loss of cervical lordosis may be associated with abnormalities in cervical muscular contractions, such as reduced muscle activation of the upper trapezius and rhomboids, and it may also provoke overfiring in the upper trapezius muscle.

### Supplementary Information

**Additional file 1: Supplementary Table 1.** The channels for both trials and the anatomical locations of the attachment site of surface electromyography electrodes.

## Data Availability

The datasets used and/or analysed during the current study are available from the corresponding author after de-identification upon request.

## Abbreviations

LL: Loss of lordosis
mCM: Modified Cobb’s method
PTM: Posterior tangent method
S-EMG: Surface electromyography
RMS: Root mean square
MVIC: Maximum voluntary isometric contraction
