# Supplementary material for: Analysis of abnormal muscle activities in patients with loss of cervical lordosis: a cross-sectional study
Source: BMC Musculoskelet Disord. 2023 Aug 22;24:666. doi: 10.1186/s12891-023-06782-3 (PMC10464463; doi:10.1186/s12891-023-06782-3)
Supplement: Supplementary file 1 — Additional file 1: Supplementary Table 1. The channels for both trials and the anatomical locations of the attachment site of surface electromyography electrodes. [file 12891_2023_6782_MOESM1_ESM.docx]

Supplementary Table 1. The channels for both trials and the anatomical locations of the attachment site of surface electromyography electrodes

| Trial | Ch. | Muscle | Locations |
| --- | --- | --- | --- |
| 1 | 1, 2 | Bil. splenius capitis | Posterior to SCM muscle at C3 level |
|  | 3, 4 | Bil. lower semispinalis cervicis | 2cm lateral from C7 spinous process |
|  | 5, 6 | Bil. sternocleidomastoid muscle | Mid-belly of sternocleidomastoid muscle |
|  | 7, 8 | Bil. upper trapezius/ levator scapulae | 2cm medial and 4cm superior area from the medial angle of scapular |
| 2 | 1, 2 | Bil. upper splenius cervicis | Posterior to SCM in C5 level |
|  | 3, 4 | Bil. upper semispinalis cervicis | 2cm lateral from C5 spinous process |
|  | 5, 6 | Bil. lower splenius cervicis | 4cm lateral from C7 spinous process |
|  | 7, 8 | Rt. middle trapezius/ rhomboids | 1cm medial from the midline medial border of scapular |
